# Advancing research translation in addiction and pain: A portfolio analysis of the NIH HEAL initiative

**DOI:** 10.1017/cts.2025.68

**Published:** 2025-04-14

**Authors:** Ginnie Sawyer-Morris, Merve Ulukaya, Bryce Kushmerick-McCune, Kendra J. Clark, Jacqueline Bruce, Scott Gatzke, Scott T. Walters, Faye S. Taxman

**Affiliations:** 1 Friends Research Institute, Baltimore, MD, USA; 2 George Mason University, Schar School of Policy and Government, Fairfax, VA, USA; 3 Sam Houston State University Department of Criminal Justice and Criminology, Huntsville, TX, USA; 4 Oregon Social Learning Center, Eugene, OR, USA; 5 University of Wisconsin Center for Health Enhancement Systems Studies, Madison, WI, USA; 6 University of North Texas Health Science Center at Fort Worth, Fort Worth, TX, USA

**Keywords:** Translational science, CFIR, implementation science, addiction, pain

## Abstract

**Background:**

To date, the NIH Helping to End Addiction Long-term (HEAL) Initiative has funded over 1,000 projects that aim to identify new therapeutic targets for pain and substance use disorder (SUD), develop nonpharmacological strategies for pain management, and improve overdose and addiction treatment across settings. This study conducted a portfolio analysis of HEAL’s research to assess opportunities to advance translation and implementation.

**Methods:**

HEAL projects (FY 2018–2022) were classified into early (T0–T1) and later (T2–T4) translational stages. Eleven coders used a 54-item data collection tool based on the Consolidated Framework for Implementation Research (CFIR) to extract project characteristics (e.g., population, research setting) relevant to translation and implementation. Descriptive statistics and visualization techniques were employed to analyze and map aggregate characteristics onto CFIR’s domains (e.g., outer setting).

**Results:**

HEAL’s portfolio comprised 923 projects (33.7% T0–T1; 67.3% T2–T4), ranging from basic science (27.1%) and preclinical research (21.4%) to clinical (36.8%), implementation (27.1%), and dissemination research (13.1%). Most projects primarily addressed either addiction (46.3%) or pain (37.4%). Implementation-related gaps included the underrepresentation of certain populations (e.g., sexual/gender minorities: 0.5%). T0–T1 projects occurred primarily in laboratory settings (35.1%), while T2–T4 projects were concentrated in healthcare settings (e.g., hospitals: 21.6%) with limited transferability to other contexts (e.g., community: 12.9%).

**Conclusion:**

Opportunities to advance translational and implementation efforts include fostering interdisciplinary collaboration, prioritizing underserved populations, engaging with community leaders and policy stakeholders, and targeting evidence-based practices in nonclinical settings. Ongoing analyses can guide strategic investments to maximize HEAL’s impact on substance use and pain crises.

## Introduction

Over the last 50 years, research on opioid use and treatments has propelled changes in policy and practice. However, substantial lags persist in the translation of addiction and pain treatments [1], with far-reaching implications for public health and individual well-being. Nearly 60 million Americans live with chronic pain, including 21 million with high-impact pain [2], and 48.5 million people with substance use disorder (SUD), including 28.9 million with alcohol use disorder (AUD), 27.2 million with drug use disorder (DUD), and 7.5 million with both [3].

Translational research in pain and addiction aims to bridge the gap between basic scientific discoveries and tangible benefits for individuals and communities affected by pain and SUDs, ultimately improving outcomes and reducing the societal burden of addiction morbidity and mortality [4–6]. Across all diseases, it takes an average of 17 years for innovations to reach patients, with only 14% of scientific knowledge ultimately being implemented in clinical practice [1]. However, the fields of addiction and pain face distinct translational challenges. In addiction research, systemic and structural barriers often delay implementation even after treatments are proven effective. For example, buprenorphine, an FDA-approved medication for opioid use disorder (OUD), took 56 years from discovery to mainstream adoption [7]. Even now, disparities in access persist for underserved populations (i.e., rural populations, racial/ethnic minority populations) due to fragmented care systems and provider shortages [8]. In pain research, the challenges occur earlier in the translational pipeline (i.e., basic science, preclinical research stages), with few novel therapeutics reaching patients in recent years [9]. Key barriers include low Phase I clinical trial success rates (pain: 0.7% success vs. other diseases: 6.5% success) [9], limited validity of preclinical models [10,11], and an overreliance on opioids despite calls for therapeutic diversification [12].

**Figure 1.**
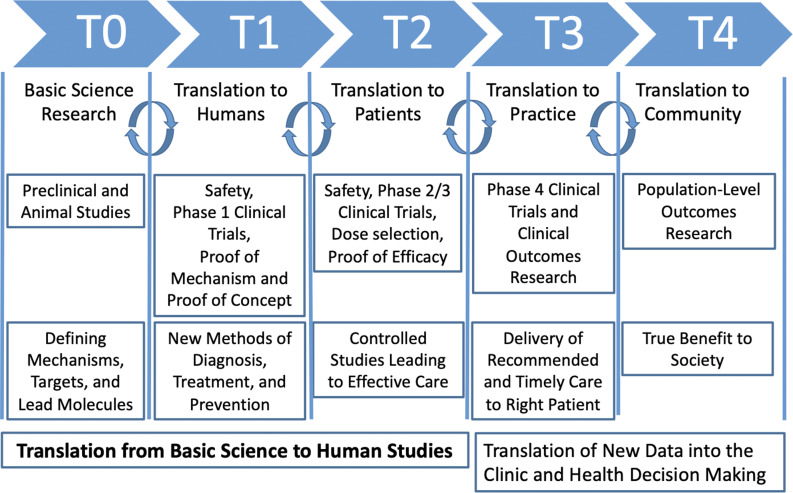
Visualization of translational stages of science (T0–T4; adapted from Blumberg et al., 2012; Goodin, 2023).

In 2018, the National Institutes of Health launched the Helping to End Addiction Long-term® (HEAL) Initiative to address these translational challenges and find scientific solutions for the public health emergency related to the opioid crisis [9]. Between 2018 and 2022, HEAL’s $3 billion investment funded nearly 1,000 projects across sectors (research, policy, public health) and translational stages (i.e., basic science to implementation science) [10]. HEAL’s pain portfolio seeks to explore public health priorities such as the fundamental mechanisms of pain and identify new targets for treatment; to develop safer, nonaddictive pain medications; and to investigate behavioral aspects of pain and develop non-pharmacological interventions, as examples. HEAL’s addiction portfolio ranges from basic brain science to population studies. HEAL-funded substance use projects include large-scale clinical trials evaluating the effectiveness of OUD medications with underserved populations, studies examining the impact of integrated pain and addiction treatment models, initiatives to train healthcare providers to be aware of and address stigma in clinical settings; and multi-state efforts to reduce overdose deaths by implementing evidence-based practices. The NIH HEAL Initiative brought together disciplines to provide multifaceted solutions that span all translational research stages (see Figure 1), including basic scientific discovery (T0), pharmacological development (T1), clinical research (T2), implementation of evidence-based treatments (T3), and community engagement and dissemination (T4) [4]. However, no systematic analysis has examined how HEAL’s portfolio addresses translational gaps across both fields.

### How scientific portfolio analyses can help advance research translation

A scientific portfolio analysis provides a macro-level perspective that can be essential for strategic planning, resource allocation, and ensuring a balanced approach to tackling various aspects of health issues [13]. It provides information about the robustness of a portfolio and answers questions about the state of scientific knowledge across key areas. Portfolio analyses can also help improve public transparency by building trust between the scientific community and the public, potentially enhancing credibility and support for research initiatives [14]. Methodologically, a portfolio analysis assesses and maps research in a funding pipeline while identifying gaps and opportunities to advance translation. Findings can help facilitate collaboration and coordination among researchers, institutions, and funding bodies, by reducing redundant efforts and promoting synergies [15,16]. A closer examination of ongoing research in the addiction and pain pipeline can assist with a better understanding of the extent of the “bench-to-trench” problem [17] and the underutilization of scientific knowledge in clinical practice [18].

### Current study

The current study presents a portfolio analysis [13] of HEAL abstracts using the translational research stages advanced by Blumberg et al [4]. and the CFIR implementation framework [17] to identify translational gaps, as well as opportunities for knowledge-building, transfer, and utilization. The portfolio analysis was conducted in two stages. First, a secondary analysis of abstracts from funded HEAL grants was conducted to identify inclusion/exclusion criteria, establish a core sample of data, and classify translational stages. Next, a custom CFIR tool was developed in Qualtrics and used to code the abstracts to identify key topics, settings, and target populations. Findings will help elucidate the translational process from basic science research to its application in clinical and community settings, enhancing our understanding of how addiction and pain research can be effectively translated into practice.

## Materials and methods

### Guiding frameworks

#### Translational research framework

The framework advanced by Blumberg et al [4]. consists of five distinct stages of translational research (T0–T4) where innovations are moved from laboratory bench to patient bedside (see Figure 1).

***Basic scientific discovery (T0) and translation to humans (T1)***. In Blumberg’s model, the first two stages (T0–T1) focus on basic scientific discovery and preclinical research to understand biological processes, disease mechanisms, and therapeutic targets [19]. T0 research typically occurs in laboratory settings, involves experimental techniques using cell cultures and animal models [20], and yields insights into neurobiological pathways, genetic factors related to addiction and pain, and potential therapeutic targets for further study. In T1, Phase I clinical trials are conducted with healthy volunteers and assess the safety, feasibility, and initial efficacy of new interventions, such as novel pharmacological agents [21]. Outputs include preliminary evidence of safety, dosage requirements, and early indications of effectiveness in humans, providing a basis for further clinical development and application [21].

***Translation to patients (T2), practice (T3), and community (T4).*** T2 focuses on translating T0–T1 findings to patients using large-scale clinical research trials to evaluate the efficacy of interventions. Research at this stage typically consists of Phase II and Phase III clinical trials with patients to assess the efficacy, optimal dosing, and safety profiles of interventions [22]. T2 outputs include data on treatment efficacy and evidence to guide clinical decision-making [23]. In T3, the emphasis shifts to integrating effective interventions into clinical practice through health services research and comparative effectiveness studies [24]. This phase may also include Phase IV trials to identify side effects or long-term outcomes [25]. T3 outputs include clinical guidelines, treatment protocols, and evidence-based health policies that optimize the delivery and impact of addiction interventions in healthcare settings. Lastly, T4 extends findings from T3 to public health systems, shifting from clinical interventions to disease management and prevention [24]. Strategies include disseminating effective interventions and promoting health equity, with outputs focused on public health interventions and policies to reduce addiction prevalence and improve population health.

#### Consolidated framework for implementation research (CFIR)

The CFIR provides a taxonomy of implementation determinants (i.e., domains, constructs) that can be used to identify barriers and facilitators to implementation and translational research [17]. The five domains of the CFIR include: Innovation Characteristics, Inner Setting, Outer Setting, Characteristics of Individuals, and Process of Change. *Innovation Characteristics* include features of the innovation, such as complexity, adaptability, and strength of evidence. The *Inner Setting* domain includes organizational factors such as culture, leadership engagement, and readiness for change. The *Outer Setting* [17] domain includes external factors that can influence implementation, including patient needs, resources, and external policies. The *Characteristics of Individuals* domain includes individual traits and perceptions of stakeholders involved in the implementation. Finally, the *Process of Change* domain includes strategies and actions used to implement the innovation, including planning, engaging stakeholders, and reflecting on progress.

### Data sources

NIH program officials provided the research team with administrative data (e.g., application ID, project number, title) for HEAL projects funded during fiscal years (FY) 2018–2022 (*N* = 1,526). Application IDs were used to query NIH Reporter to identify project abstracts. Project grant numbers and names of investigators (e.g., PI, Co-I) were used to query clinicaltrials.gov and PubMed to identify protocol papers and concept papers associated with HEAL-funded projects during FY18–FY22. The abstracts and papers (when available) served as the primary data sources for the portfolio analysis.

### Secondary analysis of NIH administrative data

**Figure 2.**
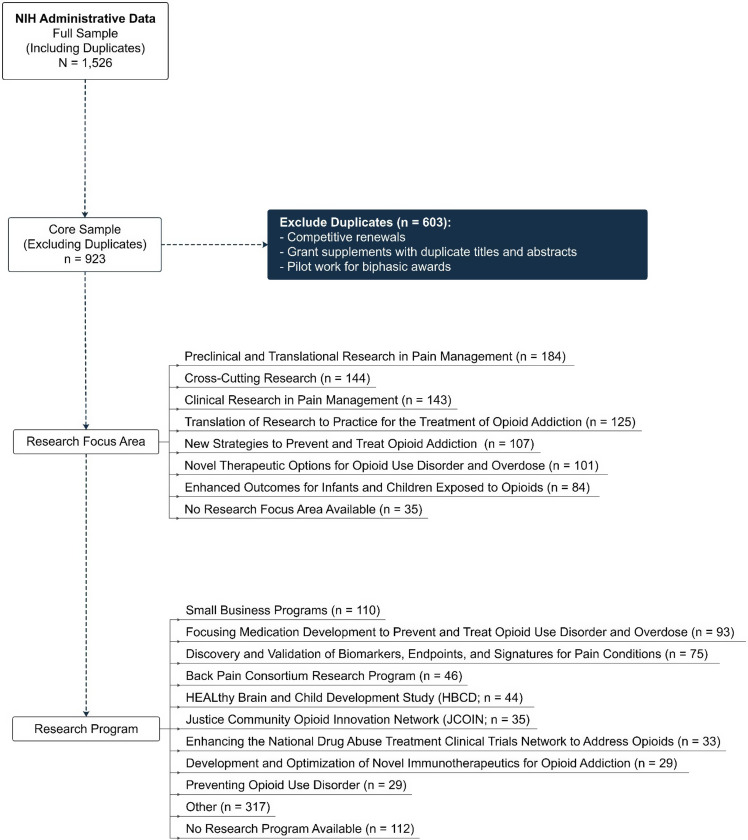
Consort diagram visualizing inclusion/Exclusion criteria for portfolio analysis.

To identify inclusion/exclusion criteria and establish a core sample of data, a secondary analysis of NIH administrative data was conducted. All abstracts (*N* = 1,526) were reviewed by two project managers to establish exclusion criteria, which included competitive renewals, grant supplements with duplicate titles and abstracts, and pilot work for biphasic awards. NIH biphasic award mechanisms (R21/R33, R61/R33, R41/R42, R43/R44, UH2/UH3, and UG3/UH3) are milestone-driven and offer an accelerated path to additional funding, if a project meets its goals within the established timelines. After excluding competitive renewals, grant supplements with duplicate titles and abstracts, and pilot work for biphasic awards, a core sample of 923 HEAL projects remained (see Figure 2).

### Coding translational stage

The two project managers reviewed the core sample of HEAL abstracts (*n* = 923) and coded the projects into two translational categories (T0–T1 and T2–T4) in Excel. Basic science, preclinical research, and phase 1 clinical trials were coded as T0–T1 (32.7%, *n* = 302 projects); projects that included translation to patients, practice, or communities were coded as T2–T4 (67.3%, *n* = 621 projects). The translational codes were imported into Stata and transformed into binary variables (T01 = 1 if the project was coded as T0–T1; T01 = 0 if the project was coded as T2–T4) and then merged with the master data (*n* = 923) using application ID.

### CFIR data collection tool

A codebook was created based on a detailed analysis of CFIR domains (e.g., outer setting, inner setting), coding guidelines, and the relevant literature [26–28] to inform the development of a 54-item data collection tool designed in Qualtrics. The two project managers, under the oversight of the project lead (PI Taxman), collaborated to develop and refine the CFIR Qualtrics tool. They met weekly with the project lead to discuss the development of survey items, refine response categories, and review coding results based on piloting the tool. Areas of disagreement were discussed until a consensus was reached and a training protocol finalized. The piloting process revealed that a subset of the CFIR survey questions (i.e., outer setting, process of change) were not relevant to T0–T1 projects. To minimize the likelihood of explainable missing data (i.e., data not missing at random), two versions of the survey were developed using the translational categories: one for T0–T1 projects and one for T2–T4 projects. For T0–T1 projects, only questions related to innovation characteristics and inner setting (e.g., population, research setting) were displayed. For T2–T4 projects, questions related to implementation characteristics (e.g., outer setting features, process of change features) were displayed in addition to innovation characteristics and inner setting features. Once the two versions were finalized, a team of nine researchers was trained to code projects using the CFIR tool. Training included three information sessions, case assignments (completed by both trainers and researchers), and an evaluation session where areas of disagreement were reviewed and discussed. Researchers began coding independently after attaining 90% consensus with the trainers.

### Data collection

Eleven researchers, including the two trainers, coded 923 HEAL-funded abstracts and papers using the CFIR tool between June 2023 and October 2023. The trainers held monthly progress meetings to share updates, obtain feedback, and address questions. Once coding was complete, data were exported from Qualtrics and cleaned in Stata 18.0.

### Descriptive portfolio analyses

Once the data from each coding phase were cleaned and merged, descriptive statistics (frequencies, percentages) were employed to describe the overall sample in terms of relevant CFIR domains (e.g., innovation characteristics, inner setting, outer setting). The T0–T1 and T2–T4 classifications were used to create aggregate, descriptive translational profiles (see Table 1). Bivariate comparisons (Pearson’s Chi-square) were conducted to explore differences between translation phases (T0–T1 vs. T2–T4) in terms of innovation characteristics (e.g., population) and inner setting (e.g., research setting). This approach highlights critical differences in the translational and implementation features of early-phase (T0–T1) and later-phase (T2–T4) projects. Table 1 shows the results organized along CFIR’s domains. Percentages reported in the text are not mutually exclusive unless otherwise specified.

**Table 1.** Summary statistics of the NIH HEAL portfolio organized along CFIR domains: overall project characteristics

Overall project characteristics	Total	T0-T1	T2-T4
	*N* = 923	*N* = 302	*N* = 621
Research focus area †			
Preclinical and translational research in pain management	184 (19.9%)	155 (51.3%)	29 (4.7%)
Novel therapeutic options for opioid use disorder and overdose	101 (10.9%)	73 (24.2%)	28 (4.5%)
Cross-cutting research	144 (15.6%)	53 (17.5%)	91 (14.7%)
New strategies to prevent and treat opioid addiction	107 (11.6%)	9 (3.0%)	98 (15.8%)
No research focus area available	35 (3.8%)	12 (4.0%)	23 (3.7%)
Enhanced outcomes for infants and children exposed to opioids	84 (9.1%)	0	84 (13.5%)
Translation of research to practice for the treatment of opioid addiction	125 (13.5%)	0	125 (20.1%)
Clinical research in pain management	143 (15.5%)	0	143 (23.0%)
Administering Institute			
NIDA	432 (46.8%)	95 (31.5%)	337 (54.3%)
NINDS	164 (17.8%)	115 (38.1%)	49 (7.9%)
NIAMS	64 (6.9%)	7 (2.3%)	57 (9.2%)
NCCIH	48 (5.2%)	5 (1.7%)	43 (6.9%)
Contact PI			
Clauw, Daniel J^1^	10 (1.1%)	0	10 (1.6%)
Bann, Carla M^1^	6 (0.7%)	0	6 (1.0%)
Bart, Gavin^1^	6 (0.7%)	0	6 (1.0%)
Brinkley-Rubinstein, Lauren^1^	6 (0.7%)	0	6 (1.0%)
Khanna, Rajesh^1^	6 (0.7%)	6 (2.0%)	0
Benjamin, Daniel K.^1^	5 (0.5%)	0	5 (0.8%)
Sowa, Gwendolyn A^1^	5 (0.5%)	0	5 (0.8%)
Comer, Sandra D^1^	4 (0.4%)	2 (0.7%)	2 (0.3%)
D’onofrio, Gail^1^	4 (0.4%)	0	4 (0.6%)
Doorenbos, Ardith Z^1^	4 (0.4%)	0	4 (0.6%)
Fair, Damien A^1^	4 (0.4%)	0	4 (0.6%)
Feinberg, Judith^1^	4 (0.4%)	0	4 (0.6%)
Fritz, Julie M^1^	4 (0.4%)	0	4 (0.6%)
Gereau, Robert W^1^	4 (0.4%)	4 (1.3%)	0
Lotz, Jeffrey C.^1^	4 (0.4%)	0	4 (0.6%)
Merhar, Stephanie L^1^	4 (0.4%)	0	4 (0.6%)
Morone, Natalia E.^1^	4 (0.4%)	0	4 (0.6%)
Renthal, William Russell^1^	4 (0.4%)	4 (1.3%)	0
Taxman, Faye S^1^	4 (0.4%)	0	4 (0.6%)
Organization			
University of Pittsburgh At Pittsburgh^1^	31 (3.4%)	7 (2.3%)	24 (3.9%)
University of Michigan At Ann Arbor^1^	27 (2.9%)	0	27 (4.3%)
Johns Hopkins University^1^	23 (2.5%)	7 (2.3%)	16 (2.6%)
Duke University^1^	22 (2.4%)	4 (1.3%)	18 (2.9%)
Yale University^1^	22 (2.4%)	1 (0.3%)	21 (3.4%)
Research Triangle Institute^1^	20 (2.2%)	2 (0.7%)	18 (2.9%)
University of California, San Francisco^1^	18 (2.0%)	2 (0.7%)	16 (2.6%)
University of Washington^1^	18 (2.0%)	4 (1.3%)	14 (2.3%)
Washington University^1^	16 (1.7%)	10 (3.3%)	6 (1.0%)
New York University School of Medicine^1^	14 (1.5%)	1 (0.3%)	13 (2.1%)
Stanford University^1^	13 (1.4%)	3 (1.0%)	10 (1.6%)
George Mason University^1^	12 (1.3%)	0	12 (1.9%)
Massachusetts General Hospital^1^	12 (1.3%)	2 (0.7%)	10 (1.6%)
National Center For Advancing Translational Sciences^1^	12 (1.3%)	12 (4.0%)	0
Boston Medical Center^1^	11 (1.2%)	0	11 (1.8%)
University of Utah^1^	11 (1.2%)	2 (0.7%)	9 (1.4%)
Brigham And Women’s Hospital^1^	10 (1.1%)	8 (2.6%)	2 (0.3%)
University of Chicago^1^	10 (1.1%)	1 (0.3%)	9 (1.4%)
University of New Mexico Health Scis Ctr^1^	10 (1.1%)	4 (1.3%)	6 (1.0%)
University of Texas Hlth Science Center^1^	10 (1.1%)	8 (2.6%)	2 (0.3%)
University of Wisconsin-Madison^1^	10 (1.1%)	2 (0.7%)	8 (1.3%)
Fiscal year †			
2018	330 (35.8%)	90 (29.8%)	240 (38.8%)
2019	223 (24.2%)	84 (27.8%)	139 (22.5%)
2020	168 (18.2%)	58 (19.2%)	110 (17.8%)
2021	132 (14.3%)	47 (15.6%)	85 (13.7%)
2022	68 (7.4%)	23 (7.6%)	45 (7.3%)
Grant type			
U grant	447 (48.4%)	126 (41.7%)	321 (51.7%)
R grant	420 (45.5%)	155 (51.3%)	265 (42.7%)
K grant^1^	8 (0.9%)	0	8 (1.3%)
Other grant	46 (5%)	21 (7%)	25 (4%)
Target of intervention †			
SUD	427 (46.3%)	74 (24.5%)	353 (56.8%)
Pain	345 (37.4%)	167 (55.3%)	178 (28.7%)
Pain and SUD	90 (9.8%)	37 (12.3%)	53 (8.5%)
Other	31 (3.4%)	14 (4.6%)	17 (2.7%)
Unknown/not specified	30 (3.3%)	10 (3.3%)	20 (3.2%)
Region †			
South	296 (32.7%)	86 (30.0%)	210 (33.9%)
Northeast	259 (28.6%)	84 (29.3%)	175 (28.3%)
West	229 (25.3%)	81 (28.2%)	148 (23.9%)
Midwest	122 (13.5%)	36 (12.5%)	86 (13.9%)

*Notes.* Column percentages are reported. Variable categories that comprise less than 5% of the overall sample were excluded from the analysis unless the category was determined to be substantively meaningful despite the low response rate (e.g., primary research design & research type categories). Other grant type includes DP grants (*n* = 2), N grants (*n* = 7), OT grants (*n* = 8), P grants (*n* = 14), S grants (*n* = 3), ZIA grants (*n* = 12). NIDA = NIH National Institute on Drug Abuse; NINDS = National Institute on Neurological Disorders and Stroke; NIAMS = NIH National Institute of Arthritis and Musculoskeletal and Skin Diseases; NCCIH = NIH National Center for Complementary and Integrative Health; OUD = opioid use disorder; † Categorical variable. § Includes supplements. 1 Category was determined to be substantively meaningful to the portfolio analysis and was included despite<5% cell frequency percentage (e.g., primary research design & research type categories). N/A indicates that the CFIR survey item was not displayed for T0–T1 projects as part of the survey design. * *p* < 0.05, ** *p* < 0.01, *** *p* < 0.05.

## Results

**Figure 3.**
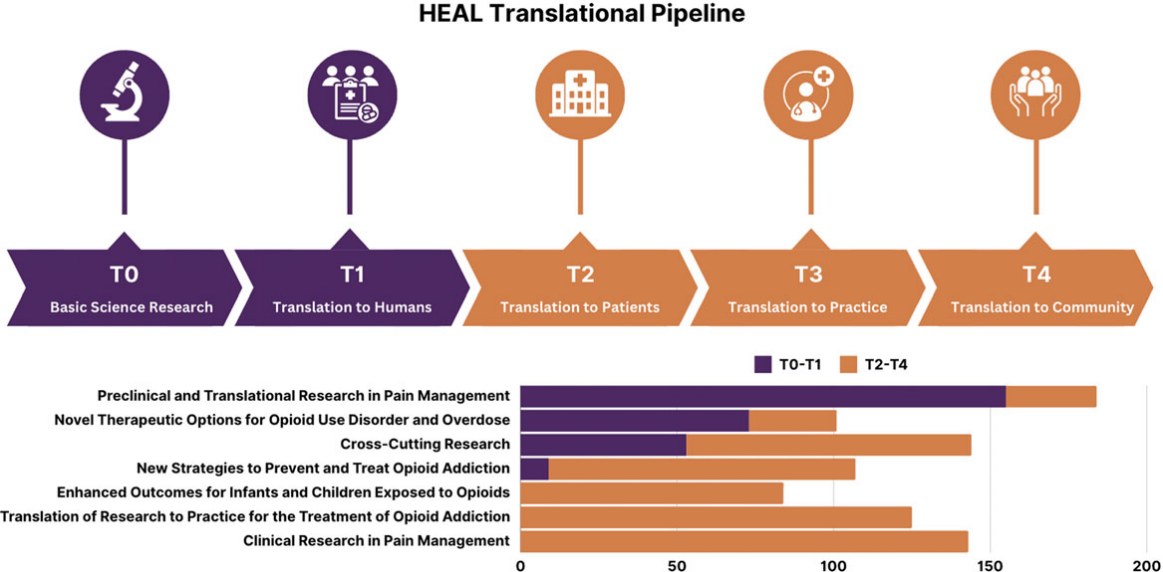
Translational diagram visualizing HEAL’s research focus areas.

Between 2018 and 2022, the HEAL portfolio comprised 923 unique projects across 19 NIH institutes and centers. These projects included 167 research centers (e.g., administrative, coordinating, data), 562 biphasic innovation grants (e.g., small business development grants, innovation grants), and 513 supplements to the original studies. The HEAL Initiative designated 42 research programs across seven research focus areas that collectively span the translational spectrum. Figure 3 provides a visual map of the research focus areas and where they fell along the translational spectrum.

### Overall project characteristics

Table 1 shows the descriptive characteristics of the research portfolio. Across the 923 projects, 162 unique organizations and 1,036 PIs (including MPIs) are represented. The primary grant types funded across the portfolio were R grants (45.5%, *n* = 420) and U grants (48.4%, *n* = 447). In terms of region, the portfolio was similarly distributed across the South (32.7%), Northeast (28.6%), and West (25.3%) with fewer projects funded in the Midwest (13.5%, *n* = 122).

### T0–T1 projects

**Figure 4.**
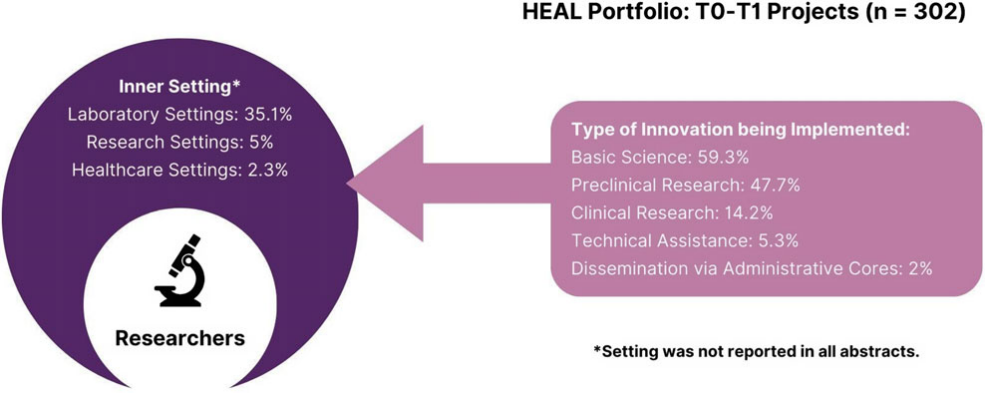
CFIR portfolio map visualizing inner setting and innovation characteristics for T0–T1 projects (Adapted from Damschroder et al., 2022).

**Figure 5.**
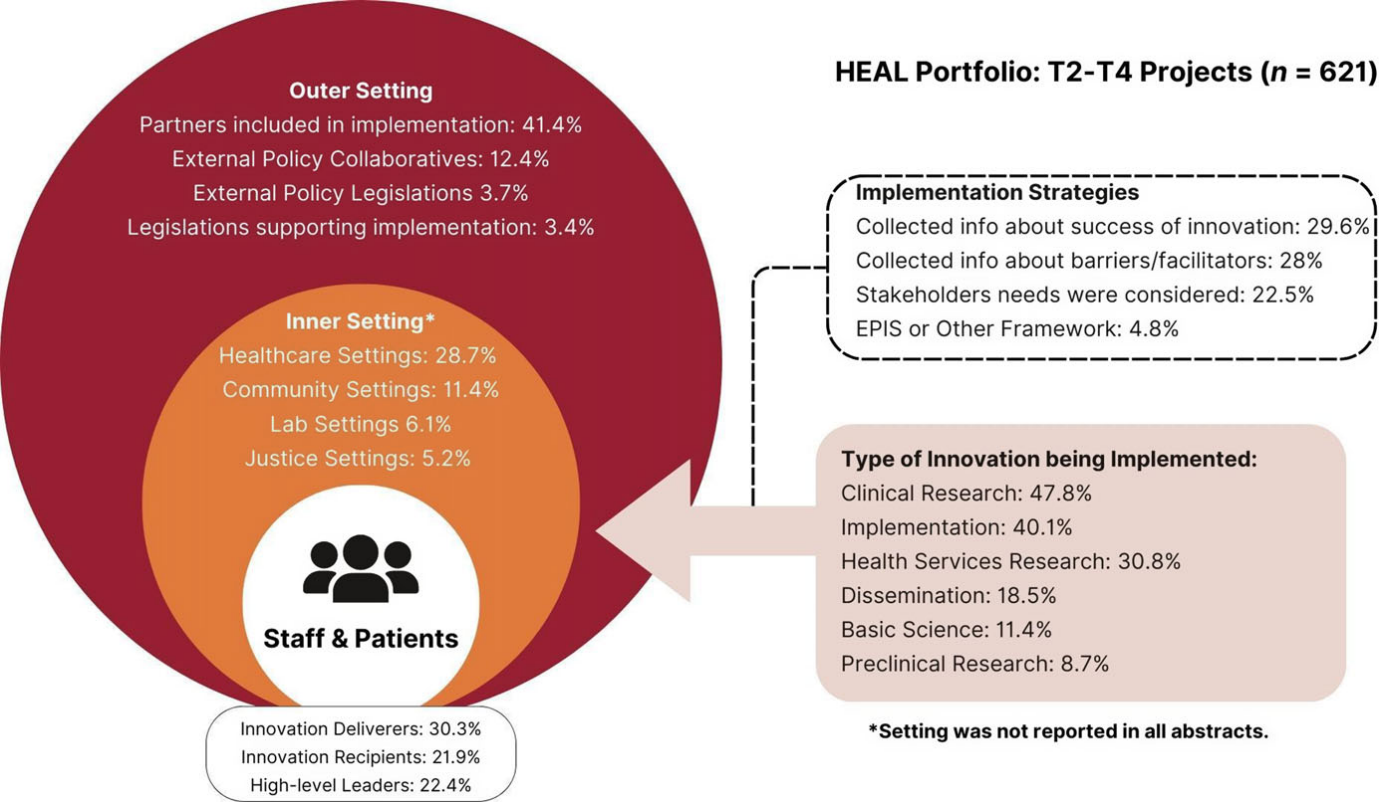
CFIR portfolio map visualizing outer setting, inner setting, process of change, and innovation characteristics for T2–T4 projects (Adapted from Damschroder et al., 2022).

Results from the descriptive analyses were used to inform two CFIR portfolio maps outlining key innovation and implementation characteristics, as well as a descriptive statistics table (see Table 2), all organized along CFIR’s domains. Separate portfolio maps were prepared for T0–T1 projects (see Figure 4) and T2–T4 projects (see Figure 5).

**Table 2.** Descriptive characteristics of HEAL portfolio presented along CFIR domains: innovation characteristics, inner setting, outer setting, and process of change domains

Innovation characteristics	Total	T0–T1	T2–T4	Test statistic	p-value
	*N* = 923	*N* = 302	*N* = 621		
Population					
Underserved populations	301 (32.6%)	55 (18.2%)	246 (39.6%)	Chi2(1) = 42.35	< .001***
Underrepresented racial/ethnic group	67 (7.3%)	1 (0.3%)	66 (10.6%)	Chi2(1) = 32.00	< .001***
Women/pregnant women	65 (7.0%)	5 (1.7%)	60 (9.7%)	Chi2(1) = 19.90	< .001***
Child/infant	53 (5.7%)	0	53 (8.5%)	Chi2(1) = 27.34	< .001***
Individuals with justice involvement^1^	41 (4.4%)	0	41 (6.6%)	Chi2(1) = 20.87	< .001***
Rural populations^1^	25 (2.7%)	0	25 (4.0%)	Chi2(1) = 12.50	< .001***
Sexual or gender minority populations^1^	5 (0.5%)	0	5 (0.8%)	Chi2(1) = 2.44	0.12
Individuals with disabilities^1^	3 (0.3%)	0	3 (0.5%)	Chi2(1) = 1.46	0.23
Individuals experiencing homelessness^1^	3 (0.3%)	0	3 (0.5%)	Chi2(1) = 1.46	0.23
Patients with pain	183 (19.8%)	37 (12.3%)	146 (23.5%)	Chi2(1) = 16.20	< .001***
Patients with SUD	180 (19.5%)	23 (7.6%)	157 (25.3%)	Chi2(1) = 40.39	< .001***
Youth/adolescent^1^	41 (4.4%)	0	41 (6.6%)	Chi2(1) = 20.87	< .001***
Researchers^1^	28 (3.0%)	6 (2.0%)	22 (3.5%)	Chi2(1) = 1.67	0.2
Older adults^1^	27 (2.9%)	1 (0.3%)	26 (4.2%)	Chi2(1) = 10.64	.001**
Human volunteer^1^	21 (2.3%)	7 (2.3%)	14 (2.3%)	Chi2(1) = 0.00	0.95
Practitioners^1^	17 (1.8%)	1 (0.3%)	16 (2.6%)	Chi2(1) = 5.67	.017*
Staff at clinic^1^	9 (1.0%)	0	9 (1.4%)	Chi2(1) = 4.42	.036*
Staff other^1^	9 (1.0%)	1 (0.3%)	8 (1.3%)	Chi2(1) = 1.93	0.17
Staff in justice system^1^	7 (0.8%)	0	7 (1.1%)	Chi2(1) = 3.43	0.064
Innovation source					
Innovation source: researchers	756 (81.9%)	254 (84.1%)	502 (80.8%)	Chi2(1) = 1.46	0.23
Innovation source: Practitioners/clinicians^1^	16 (1.7%)	0	16 (2.6%)	Chi2(1) = 7.92	.005**
Innovation source: policymakers^1^	6 (0.7%)	0	6 (1.0%)	Chi2(1) = 2.94	0.087
Innovation type					
Clinical research	340 (36.8%)	43 (14.2%)	297 (47.8%)	Chi2(1) = 98.52	< .001***
Basic science	250 (27.1%)	179 (59.3%)	71 (11.4%)	Chi2(1) = 235.45	< .001***
Implementation	250 (27.1%)	1 (0.3%)	249 (40.1%)	Chi2(1) = 162.69	< .001***
Preclinical research	198 (21.5%)	144 (47.7%)	54 (8.7%)	Chi2(1) = 183.28	< .001***
Health services research	191 (20.7%)	0	191 (30.8%)	Chi2(1) = 117.12	< .001***
Alcohol, tobacco, and other drugs	172 (18.6%)	37 (12.3%)	135 (21.7%)	Chi2(1) = 12.06	< .001***
Data sharing/analytics	129 (14.0%)	22 (7.3%)	107 (17.2%)	Chi2(1) = 16.72	< .001***
Dissemination	121 (13.1%)	6 (2.0%)	115 (18.5%)	Chi2(1) = 48.75	< .001***
Technical assistance	106 (11.5%)	16 (5.3%)	90 (14.5%)	Chi2(1) = 16.90	< .001***
Maternal, infant, and child health	106 (11.5%)	0	106 (17.1%)	Chi2(1) = 58.24	< .001***
Epidemiology	81 (8.8%)	0	81 (13.0%)	Chi2(1) = 43.18	< .001***
Public Health Infrastructure	79 (8.6%)	2 (0.7%)	77 (12.4%)	Chi2(1) = 35.76	< .001***
Health communication and information technology	63 (6.8%)	7 (2.3%)	56 (9.0%)	Chi2(1) = 14.34	< .001***
Social determinants of health and health disparities	56 (6.1%)	7 (2.3%)	49 (7.9%)	Chi2(1) = 11.07	< .001***
Research design					
Randomized control trial	86 (10.2%)	0	86 (15.3%)	Chi2(1) = 47.65	< .001***
Longitudinal^1^	41 (4.9%)	0	41 (7.3%)	Chi2(1) = 21.51	< .001***
Implementation effectiveness hybrid^1^	39 (4.6%)	0	39 (7.0%)	Chi2(1) = 20.41	< .001***
Observational^1^	36 (4.3%)	0	36 (6.4%)	Chi2(1) = 18.77	< .001***
Monitoring/surveillance^1^	24 (2.9%)	0	24 (4.3%)	Chi2(1) = 12.33	< .001***
Research protocol development^1^	15 (1.8%)	2 (0.7%)	13 (2.3%)	Chi2(1) = 2.74	0.098
Motivation for innovation‡					
Expand/renew prior grant research	120 (13.0%)	0	120 (19.3%)	Chi2(1) = 67.08	< .001***
Gap - DNK genetic pathways/functioning	146 (15.8%)	90 (29.8%)	56 (9.0%)	Chi2(1) = 65.91	< .001***
Need - improved OUD treatment	221 (23.9%)	61 (20.2%)	160 (25.8%)	Chi2(1) = 3.46	0.063
Need - improved pain treatment	190 (20.6%)	57 (18.9%)	133 (21.4%)	Chi2(1) = 0.80	0.37
Need - validate therapeutic pain targets	138 (15.0%)	110 (36.4%)	28 (4.5%)	Chi2(1) = 162.76	< .001***
Need - nonaddictive pain control	78 (8.5%)	61 (20.2%)	17 (2.7%)	Chi2(1) = 80.07	< .001***
Problem - drug overdose	133 (14.4%)	62 (20.5%)	71 (11.4%)	Chi2(1) = 13.63	< .001***
Problem particularly effects special population	128 (13.9%)	4 (1.3%)	124 (20.0%)	Chi2(1) = 59.12	< .001***
Problem - OUD (General)	114 (12.4%)	25 (8.3%)	89 (14.3%)	Chi2(1) = 6.88	.009**
Problems with current pain Tx	108 (11.7%)	61 (20.2%)	47 (7.6%)	Chi2(1) = 31.37	< .001***
Problem - SUD resulting from Pain Tx	107 (11.6%)	69 (22.8%)	38 (6.1%)	Chi2(1) = 55.48	< .001***
Problem - poor treatment of pain	83 (9.0%)	47 (15.6%)	36 (5.8%)	Chi2(1) = 23.68	< .001***
Problem - pain decreases quality of life	79 (8.6%)	54 (17.9%)	25 (4.0%)	Chi2(1) = 49.84	< .001***
Problem - in utero drug exposure	67 (7.3%)	0 (0.0%)	67 (10.8%)	Chi2(1) = 35.13	< .001***
Problems with current OUD Tx	56 (6.1%)	31 (10.3%)	25 (4.0%)	Chi2(1) = 13.88	< .001***
Problem - access to Tx	47 (5.1%)	1 (0.3%)	46 (7.4%)	Chi2(1) = 21.05	< .001***
Problem - retention in Tx1	39 (4.2%)	4 (1.3%)	35 (5.6%)	Chi2(1) = 9.33	.002**
Problem - environmental influences^1^	32 (3.5%)	0 (0.0%)	32 (5.2%)	Chi2(1) = 16.12	< .001***
Outcomes					
Other medical conditions treatment evaluation^1^	23 (2.5%)	5 (1.7%)	18 (2.9%)	Chi2(1) = 1.29	0.26
OUD treatment evaluation	258 (28.0%)	66 (21.9%)	192 (30.9%)	Chi2(1) = 8.29	.004**
Pain treatment evaluation	229 (24.8%)	96 (31.8%)	133 (21.4%)	Chi2(1) = 11.72	< .001***
SUD treatment evaluation^1^	45 (4.9%)	7 (2.3%)	38 (6.1%)	Chi2(1) = 6.33	.012*

Inner setting	Total	T0–T1	T2–T4		
	*N* = 923	*N* = 302	*N* = 621	Test statistic	*p*-value
Research setting					
Healthcare setting	185 (20%)	7 (2.3%)	178 (28.7%)	Chi2(1) = 88	< .001***
Lab setting	144 (15.6%)	106 (35.1%)	38 (6.1%)	Chi2(1) = 129.6	< .001***
Research setting	110 (12%)	15 (5%)	95 (15.3%)	Chi2(1) = 20.66	< .001***
Family/community settings	71 (7.7%)	0	71 (11.4%)	Chi2(1) = 37.41	< .001***
School setting^1^	7 (0.8%)	0	7 (1.1%)	Chi2(1) = 3.43	0.064
Justice setting^1^	32 (3.5%)	0	32 (5.2%)	Chi2(1) = 16.12	< .001***
Information technology infrastructure					
Reporting and analysis	N/A	N/A	169 (27.2%)		
Data management	N/A	N/A	160 (25.8%)		
Data storage	N/A	N/A	152 (24.5%)		
Electronic documentation	N/A	N/A	100 (16.1%)		
Telecommunications	N/A	N/A	84 (13.5%)		
Implementation resources					
Intervention team	N/A	N/A	119 (19.2%)		
Materials and equipment supplies	N/A	N/A	91 (14.7%)		
Staffing	N/A	N/A	75 (12.1%)		
Budget or financial resources	N/A	N/A	70 (11.3%)		
Training	N/A	N/A	67 (10.8%)		
Provider buy-in^1^	N/A	N/A	43 (6.9%)		

Outer setting	Total	T0-T1	T2-T4		
	*N* = 923	*N* = 302	*N* = 621		
Needs addressed by innovation					
Individual needs	N/A	N/A	276 (44.4%)		
Needs for physical health conditions	N/A	N/A	211 (34.0%)		
Organizational	N/A	N/A	100 (16.1%)		
Needs for mental health conditions	N/A	N/A	93 (15.0%)		
Social	N/A	N/A	66 (10.6%)		
Economic^1^	N/A	N/A	23 (3.7%)		
Housing^1^	N/A	N/A	8 (1.3%)		
Nutritional^1^	N/A	N/A	3 (0.5%)		
Type of external policy					
Collaboratives	N/A	N/A	77 (12.4%)		
Policy and regulation^1^	N/A	N/A	23 (3.7%)		
Recommendations and guidelines^1^	N/A	N/A	18 (2.9%)		
External mandates^1^	N/A	N/A	12 (1.9%)		
Other^1^	N/A	N/A	12 (1.9%)		
Public or benchmark reporting^1^	N/A	N/A	1 (0.2%)		
Partners		N/A			
Partnerships Identified	N/A	N/A	257 (41.4%)		
Role of partners					
Recruitment or retention	N/A	N/A	106 (17.1%)		
Role of partner: implementation in their agency	N/A	N/A	103 (16.6%)		
Research design	N/A	N/A	79 (12.7%)		
Data collection	N/A	N/A	73 (11.8%)		
Dissemination	N/A	N/A	67 (10.8%)		
Analyses^1^	N/A	N/A	51 (8.2%)		
Legislations/regulations supporting implementation^1^	N/A	N/A	21 (3.4%)		
Resource landscape of innovation					
Resources from collaborating agencies	N/A	N/A	60 (9.7%)		
New resources for NIH, coordinating centers, medicaid/medicare, etc.^1^	N/A	N/A	8 (1.3%)		
Medicaid expansion^1^	N/A	N/A	2 (0.3%)		

Process of change	Total	T0–T1	T2–T4		
	*N* = 923	*N* = 302	*N* = 621		
Individuals Involved in implementation					
Innovation deliverers	N/A	N/A	188 (30.3%)		
Implementation team members	N/A	N/A	148 (23.8%)		
High-level leaders	N/A	N/A	139 (22.4%)		
Innovation recipients	N/A	N/A	136 (21.9%)		
Implementation leads	N/A	N/A	114 (18.4%)		
Facilitators	N/A	N/A	85 (13.7%)		
Other implementation support^1^	N/A	N/A	27 (4.3%)		
Quan/Qual data collected about implementation					
Collected Info about recipients	N/A	N/A	174 (28.0%)		
Collected info about barriers and facilitators	N/A	N/A	103 (16.6%)		
Collected info about deliverers	N/A	N/A	90 (14.5%)		
Attracted and encouraged deliverers and recipients of innovation					
Attracted and encouraged deliverers	N/A	N/A	74 (11.9%)		
Attracted and encouraged recipient	N/A	N/A	71 (11.4%)		
Abstract identified implementation champion					
Implementation champion: Yes	N/A	N/A	154 (24.8%)		
Innovation included externally appointed individuals	N/A	N/A	72 (11.6%)		
Type of external agents: outside researchers^1^	N/A	N/A	27 (4.3%)		
Type of external agents: hired consultants^1^	N/A	N/A	12 (1.9%)		
Type of external agents: facilitators^1^	N/A	N/A	11 (1.8%)		
Type of external agents: trainers^1^	N/A	N/A	3 (0.5%)		
Strategies used to implement innovation					
Well-developed plan	N/A	N/A	178 (28.7%)		
Pilots or trials	N/A	N/A	112 (18.0%)		
Breaking down the intervention	N/A	N/A	111 (17.9%)		
Successes in early increments^1^	N/A	N/A	35 (5.6%)		
EPIS or other framework^1^	N/A	N/A	30 (4.8%)		
Dry runs^1^	N/A	N/A	11 (1.8%)		
Characteristics considered in implementation planning					
Collecting info about success of the innovation	N/A	N/A	184 (29.6%)		
Stakeholders’ needs are considered	N/A	N/A	140 (22.5%)		
Collecting info about the success of the implementation	N/A	N/A	88 (14.2%)		
Strategies appropriate for subgroups	N/A	N/A	67 (10.8%)		
Progress toward goals is tracked	N/A	N/A	41 (6.6%)		
Appropriate communication channels^1^	N/A	N/A	26 (4.2%)		
Adapting					
Study Involved Integrating Innovation into the Setting	N/A	N/A	127 (20.5%)		

*Notes.* Column percentages are reported. Variable categories that comprise less than 5% of the overall sample were excluded from the analysis unless the category was determined to be substantively meaningful despite the low response rate (e.g., primary research design and research type categories). Motivation for project codes included problems, needs, and gaps addressed by innovation. OUD = opioid use disorder; Tx = Treatment. ‡ Motivation for project codes included problems, needs, and gaps addressed by innovation. † Categorical variable. § Includes supplements. 1 Category was determined to be substantively meaningful to the portfolio analysis and was included despite<5% cell frequency percentage (e.g., primary research design and research type categories). N/A indicates that the CFIR survey item was not displayed for T0–T1 projects as part of the survey design. * *p* < 0.05, ** *p* < 0.01, *** *p* < .05.

### Innovation characteristics and inner setting

Most T0–T1 projects (32.7%, *n* = 302) were coded as basic science (59.3%, *n* = 179), preclinical research (47.7%, *n* = 144), or clinical research (14.2%, *n* = 43). A small number of projects focused on dissemination via administrative cores (2%, *n* = 6), health communication and information technology (2.3%, *n* = 7), and technical assistance (5.3%, *n* = 16). The most common outcome addressed by T0–T1 projects was efficacy (34.2%, *n* = 103), including pain treatment efficacy (*n* = 58) and OUD/SUD treatment efficacy (*n* = 45). Safety outcomes (17.6%, *n* = 53) were also frequently addressed by T0–T1 projects, including pain treatment safety (*n* = 26), OUD/SUD treatment safety (*n* = 26), and other medical conditions treatment safety (*n* = 1). Few T0–T1 projects took place in a healthcare setting (2.3%, *n* = 7). Instead, most were conducted in laboratory settings (35.1%, *n* = 106) with animal or cell models (58.6%, *n* = 177).

### T2–T4 projects

#### Innovation characteristics and inner setting

T2–T4 (*n* = 621) projects included basic research (11.4%, *n* = 71), preclinical research (8.7%, *n* = 54), clinical research (47.8%, *n* = 297), implementation (40.1%, *n* = 249), health services research (30.8%, *n* = 191), and dissemination (18.5%, *n* = 115). Several research designs were employed across T2–T4 projects including randomized control trials (15.3%, *n* = 86), epidemiological approaches (13%, *n* = 81), longitudinal designs (7.3%, *n* = 41), hybrid effective-implementation designs (7%, *n* = 39), observational studies (6.4%, *n* = 36), and research protocol development (2.3%, *n* = 13). The most common outcomes cited in T2–T4 abstracts were efficacy-related (27.1%, *n* = 168), including OUD/SUD treatment efficacy (15.6%, *n* = 97) and pain treatment efficacy (12.4%, *n* = 69). Implementation-related outcomes were also prevalent among T2–T4 projects, including acceptability, feasibility, and sustainability. Many projects were implemented in healthcare settings (28.7%, *n* = 178), including hospitals, clinics, doctor’s offices, and substance use treatment facilities (both in-patient and outpatient). Other settings included family and community settings (e.g., community-based, client-centered prevention homes; mobile harm reduction services; 11.4%, *n* = 71), justice settings (i.e., prisons, jails, and probation/parole offices; 5.2%, *n* = 32), educational settings (e.g., schools; 1.1%, *n* = 7), and laboratory settings (6.1%, *n* = 38).

Regarding motivation for the innovation or project, 41% of the portfolio abstracts indicated their work addressed an existing gap in research (e.g., disparities in treatment efficacy by population, unknown genetic pathways/functioning); 88% addressed research and practice needs (e.g., need for improved OUD treatment, need for improved pain treatment); and 80% identified problems (drug overdose). In terms of innovation target (i.e., addiction vs. pain), the portfolio included substance use-only projects (46.3%, *n* = 427), pain-only projects (37.4%, *n* = 345), and both substance use and pain projects (9.8%, *n* = 90). In terms of population, 36.6% of HEAL’s portfolio focuses on patients (*n* = 331); of these, 42% were patients with pain (*n* = 139), 46% were patients with SUD (*n* = 153), and 12% were patients with pain and SUD (*n* = 39). Nearly one in three projects in the portfolio focus on underserved populations (e.g., underrepresented racial/ethnic populations, women/pregnant women, individuals with justice involvement, rural populations), and 2.3% of projects engage human volunteers (*n* = 21).

#### Outer setting

T2–T4 innovations primarily addressed individual needs (44.4%, *n* = 276; e.g., needs of patients and providers) and needs for physical health conditions (34%, *n* = 211; e.g., nonopioid pain treatment). Projects also addressed organizational needs (16.1%; *n* = 100), needs for mental health conditions (10.6%, *n* = 66; e.g., co-occurring mental illness and SUD), and social needs (15%; *n* = 93; implicit bias; racial inequities). In some cases, external policies may have supported the innovation or project; the most commonly cited external policy was “Collaboratives” (12.4%; *n* = 77). Examples of collaboratives included the NIDA Clinical Trials Network, the Outcomes of Babies with Opioid Exposure (OBOE) Consortium, HEALing Communities, and the HEAL Back Pain Consortium (BACPAC). Over 41% of abstracts (*n* = 257) mentioned a partner; examples of partners included stakeholder engagement boards, advisory boards, medical and treatment centers, pharmaceutical companies, and policy nonprofits. Across most projects, partners acted as research sites (16.6%, *n* = 103) or were involved in recruitment or retention (17.1%, *n* = 106). Partners also helped with research design (12.7%, *n* = 79), data collection (11.8%, *n* = 73), dissemination (10.8%, *n* = 67), and analyses (8.2%, *n* = 51).

#### Implementation process domain

The implementation process domain included individuals involved with the change process, as well as implementation strategies used as part of the change process. Individuals involved with T2–T4 projects included innovation deliverers (30.3%, *n* = 188), implementation team members (23.8%, *n* = 148), high-level leaders (22.4%, *n* = 139), innovation recipients (21.9%, *n* = 136), implementation leads (18.4%, *n* = 114), and facilitators (13.7%, *n* = 85). Many projects reported collecting information about recipients of the innovation (28%, *n* = 174) either before or as part of the implementation. Fewer T2–T4 projects collected information about innovation deliverers (14.5%, *n* = 90) and barriers and facilitators to implementation (16.6%, *n* = 103).

## Discussion

Historically, it takes 17 years for scientific discoveries to reach patients and even then only 14% of scientific knowledge is utilized in clinical practice [1,18]. This translational gap is more pronounced in the addiction field due to policy and socially driven barriers like stigma, as evidenced by buprenorphine’s 56-year progression from discovery to mainstream adoption [7]. To accelerate research along the addiction and pain scientific pipeline, the NIH HEAL Initiative funded a diverse portfolio of research spanning the translational spectrum (T0–T4). Between 2018 and 2022, HEAL funded 32.7% (*n* = 302) early-phase projects (T0–T1; basic science, preclinical research) and 67.3% (*n* = 621) mid- to late-phase projects (T2–T4; clinical research and implementation projects). Findings from this portfolio analysis underscore HEAL’s investment and highlight several translational and implementation-related opportunities to enhance its impact.

As shown in this analysis, translational gaps are most pronounced at later research stages (e.g., implementation, dissemination, policy), where systemic barriers hinder equitable access to effective treatments. Legislative efforts, such as the SUPPORT for Patients and Communities Act [29] and the Mainstreaming Addiction Treatment (MAT) Act [8], have aimed to address these barriers by expanding Medicaid coverage for medication-assisted treatment and eliminating the Drug Enforcement Elimination (DEA) waiver for prescribing buprenorphine [30]. Despite these advancements, disparities in buprenorphine uptake persist, disproportionately affecting rural communities [8,31] and racial/ethnic minority populations [32–34]. While the evidence base for MOUD is well-established, no FDA-approved medications currently exist to treat stimulant and cannabis use disorders [35]. This is an opportunity for early-stage addiction research (T0–T1).

In pain research, translational gaps are concentrated at earlier stages of the research-to-practice pipeline (basic science, preclinical research),[11] and the development of novel pain therapeutics (T0) has been slow. If we exclude calcitonin gene-related peptide (CGRP) antibodies and small-molecule antagonists for the treatment of migraine, only a handful of drugs with novel targets have made it into the hands of clinicians and patients over the past 15 years, the majority of which were opioids. Specific barriers to the translation of novel pain innovations include low Phase I clinical trial success rates relative to other diseases (pain: 0.7% success vs. other diseases: 6.5% success) [9], limited validity of preclinical animal models that fail to replicate the subjective experience of clinical pain in humans [10,11], and an overreliance on opioids despite calls to diversify therapeutic options [12].

While pain is recognized as a significant factor in the pathway to opioid use and misuse, the fields of pain and addiction remain largely siloed, as shown by this analysis, further compounding translational challenges and contributing to fragmented care. Addressing these gaps requires bidirectional communication between researchers and clinicians to ensure that basic research responds to the needs of clinical practice and prioritizes interventions that integrate the management of co-occurring pain and addiction.

### Translational gaps

#### Challenges in early-stage translational research

In general, early-phase projects (T0–T1) may be particularly vulnerable to translation failure due to substantial costs ($1.1–$1.5 billion) [36,37] and lengthy timelines (up to a decade) [4] required to move basic scientific discoveries to clinical settings [4,6]. Nearly 33% of HEAL’s portfolio are T0–T1 projects and subject to these challenges. Of the 302 T0–T1 projects in HEAL’s portfolio, 67.6% target pain. In recent decades, novel pain innovations have faced specific challenges related to translation, including low Phase I clinical trial success rates relative to other diseases (pain: 0.7% success vs. other diseases: 6.5% success) [9]. To address these barriers, HEAL has invested in key resources, including data centers, research programs focused on developing pain targets and signatures (see Figure 1), and the Early Phase Preclinical Investigation Network (EPPIC-Net), which provides crucial support for regulatory submissions. However, more targeted strategies are needed. For instance, strategic networking with T2–T4 researchers can facilitate knowledge transfer, while FDA training and mentored research opportunities for early-career scientists can build the necessary expertise to navigate complex regulatory landscapes [38,39]. Additionally, enhanced funding mechanisms, interdisciplinary collaborations through public–private partnerships, and robust regulatory guidance are critical to bridging this translational gap.

#### Interdisciplinary collaboration to address both pain and addiction

Prioritizing research at the intersection of addiction and pain is crucial for developing integrated treatment approaches [40]. These two conditions, and their underlying mechanisms, are complex and interrelated yet the two fields remain largely siloed. Currently, only 9.8% of HEAL projects address both substance use and pain, indicating a substantial opportunity to enhance research in this area. Integrating research efforts and forging new collaborations across the two independent fields can ensure that findings are rapidly translated into clinical practice, benefiting patients more quickly [18]. Addressing these co-occurring conditions can lead to better management strategies, ultimately improving patient outcomes and quality of life [40].

### Implementation-related gaps

#### Implementation science and later-stage translational research

In the portfolio analysis, CFIR was used to assess implementation characteristics (e.g., inner setting, outer setting, implementation process) of HEAL’s T2–T4 projects. Many evidence-based innovations that survive early translational stages are not adequately implemented or disseminated equitably [41–44]. For later-stage (T2–T4) projects, our findings suggest a collective focus on safety, efficacy, and implementation effectiveness outcomes of both pain and addiction interventions and treatments, which include but are not limited to medications, behavioral interventions, and digital health products. The CFIR framework provides valuable insights into the determinants of successful implementation, yet our analysis revealed that many funded projects inadequately specify these critical factors in their project descriptions. For instance, 37% of the abstracts analyzed did not detail the research settings, which complicates efforts to generalize findings across different populations and contexts. This omission highlights a broader issue within later-stage translational research: the lack of specificity and transparency can impede the development of a robust knowledge base necessary for effective implementation across diverse settings.

#### Research setting diversity and impact in the HEAL initiative

The distribution of projects, as represented by their abstracts, within the NIH HEAL Initiative suggests potential trends in certain settings. Early-stage translational projects (T0–T1) appeared to predominantly take place in laboratory settings, focusing on basic science and initial testing phases. For nearly a decade, researchers have called on innovations to be more rapidly translated into clinical settings [45]. Biphasic funding mechanisms allow for this accelerated translation process wherein prototypes (i.e., T1 technology) may be tested with patients as part of a feasibility trial in Phase 1 before moving to a full RCT trial in Phase 2. Clarifying, or differentiating, the contexts in which technologies vs. medications vs. other innovations are developed and initially deployed could help characterize the stage and pace of translation. While our findings regarding T0–T1 projects and laboratory settings may seem obvious, the underlying takeaway is that “research setting” should be clarified in T0–T1 projects moving forward, especially given the rapid proliferation of digital treatment innovations.

In contrast, mid-to-late-stage translational projects (T2–T4) were more commonly conducted in healthcare settings (28.7%) such as doctors’ offices, hospitals, and substance use treatment clinics, suggesting a shift towards clinical application and implementation. Justice (5.2%), education (1.1%), and community settings (11.4%) were less frequently represented in T2–T4 studies. While these findings highlight potential research setting trends, it is important to note that these findings are derived from abstract-level information, which may not fully capture the diversity or complexity of project settings. To enhance the reach and impact of HEAL-supported research, further investments could consider increasing investments in underrepresented settings (e.g., justice) and diversifying implementation environments (e.g., faith-based organizations, schools). Such efforts may facilitate broader engagement with multiple populations and communities.

### Population focus: addressing disparities in research representation

While approximately 32% of the portfolio focused on underserved populations, there was significant variation in representation across different groups. Sexual or gender minority populations (0.5%), individuals with disabilities (0.3%), and individuals experiencing homelessness (0.3%) were notably underrepresented compared to other demographic groups. These populations face disproportionate risks of SUD, yet their inclusion in both this portfolio and broader research initiatives remains limited. Strengthening research initiatives aimed at these groups holds the potential to address long-standing health disparities and improve treatment outcomes equitably.

#### Sexual and gender minority populations

Sexual and gender minority (SGM) communities were underrepresented within HEAL’s portfolio, accounting for only 0.5% of funded projects. This disparity underscores the need for targeted research that considers the unique biological, social, and psychological factors influencing health outcomes in these communities. SGM populations [46] face several challenges in accessing treatment, including providers’ negative or ambivalent views about SGM patients and the lack of substance use programs tailored to the needs of SGM populations [47–49]. Moreover, SGM individuals are more likely to anticipate discrimination or rejection in treatment, such as providers’ bias and the safety of identity disclosure [50]. Cross-sector investments are needed to reach SGM communities and address the complex interplay of factors faced by this population. For instance, JCOIN’s upcoming HIV initiative represents a promising opportunity to prioritize and expand engagement with SGM and justice-involved populations. This strategic, cross-sector focus could catalyze efforts to mitigate health disparities and enhance treatment equity.

#### Individuals with disabilities

Both pain-related disorders and SUDs are leading causes of global disability [49,50]. Chronic pain conditions such as low back pain and headache disorders, alongside HIV/AIDS, DUDs, and AUDs, rank among the top 20 leading causes of global disability [51]. Despite the significant overlap between chronic pain, addiction, and disabilities, individuals with disabilities are underrepresented in HEAL-funded research (0.3% of the portfolio). This gap may stem from a lack of explicit framing rather than a deficiency in HEAL’s portfolio. Given the multisector focus on pain management and addiction treatment, it is likely that HEAL *is* funding early- and later-stage translational research with important implications for individuals with disabilities (e.g., migraine-related pain studies, neuropathic pain targets, chronic low back pain interventions). Integrating a disabilities-related perspective into HEAL-funded research could not only help acknowledge the substantial overlap between chronic pain, addiction, and disabilities but also ensure that interventions and treatments are inclusive and effective for individuals with diverse health needs. Moreover, by adopting disability-related language and frameworks across the translational spectrum, HEAL could enhance the relevance and applicability of its research findings within the context of existing legal protections such as the Americans with Disabilities Act [52]. This approach would further compliance with disability rights legislation and promote equity in healthcare access and outcomes.

#### Individuals experiencing homelessness

Homelessness exacerbates the difficulties in managing pain and addiction due to financial insecurity, stagnant wages, disabilities, rising housing costs, and declining affordable housing options [53,54]. Despite heightened vulnerability, only 0.3% of HEAL-funded projects focus on individuals experiencing homelessness. Recent legal developments, such as the Supreme Court’s decision in City of Grants Pass, Oregon v. Johnson [55], have highlighted the constitutional challenges of criminalizing homelessness without providing adequate shelter and support services and underscored the urgency of addressing homelessness within public health frameworks. In their annual report, the HEAL Initiative emphasized a whole-person and integrated approach to science that recognizes the “multifaceted biological, psychological, social, and environmental dimensions” of addiction and pain [56]. Such an approach considers the socioeconomic determinants of health that disproportionately affect homeless populations. Investing in community-based participatory research and partnerships with housing organizations can help develop and implement comprehensive interventions. Furthermore, targeted funding mechanisms and pilot programs that integrate housing stability with addiction and pain management services are crucial. By expanding the research portfolio to include a greater focus on homelessness, HEAL can address critical health disparities and improve outcomes for one of the most marginalized groups in society.

### Expanding the reach

Recently, researchers have envisioned a sixth phase (T5) of the translational continuum that engages policymakers and scientists to define the path that maximizes investments in system-wide and equitable implementation of evidence-based treatments [24]. Historically, translational research has faced significant barriers to widespread adoption, including regulatory hurdles, inadequate funding for large-scale implementation, and persistent stigma, which is particularly hard-felt in the fields of addiction and pain management. The proposed T5 stage addresses these challenges by fostering collaboration between researchers, policymakers, and community stakeholders to create robust frameworks for dissemination. A key component of T5 is the proactive engagement with policy and decision-makers at local, state, and federal levels to align research findings with health policy and funding priorities. This involves translating scientific evidence into policy briefs, guidelines, and advocacy efforts that can influence legislation and healthcare practices. The need for sustainable, accurate, and open-source measurement of implementation determinants (e.g., population, setting) becomes critically important at this stage. One way HEAL can improve ongoing measurement of these outcomes is by employing a structured abstract as part of the application process, requiring, or at least encouraging investigators, to report on key determinants like research settings, implementation partners (if applicable), and population in the project summary of their grant application. Integrating broad-scale measurement and implementation with strategic policy partnerships and efforts can help HEAL further bridge the gap between clinical innovation and real-world application, ensuring that scientific advances are accessible and beneficial to all segments of society.

### Limitations

This analysis provides a high-level overview of HEAL’s research portfolio, highlighting advancements and areas for future investment. Further analysis may be necessary for in-depth insights at the program level. Despite efforts to ensure data validity (e.g., interrater reliability testing, training sessions, coding meetings), some subjectivity in coding abstracts remains. Additionally, NIHReporter abstracts, which do not include all project elements, were the primary data source. When available, coders also reviewed supplementary material (e.g., concept papers). Grant abstracts are relatively short – no more than 30 lines of text – and cannot contain the full details of a project. As a result, much granularity about the project is lost with only the abstract as the input. However, abstracts are publicly available and representative of the scientific portfolio, making them appropriate for a portfolio analysis. These factors should be considered when drawing conclusions about the portfolio.

### Conclusions

The NIH HEAL Initiative has played a pivotal role in addressing the opioid crisis, especially amid challenges posed by COVID-19. However, several opportunities to advance translation and implementation remain. This portfolio analysis explored HEAL’s 2018–2022 research portfolio using Blumberg’s translational stages and the CFIR. Our findings highlight opportunities for HEAL to enhance its impact through strategic research initiatives and collaborative efforts between research, practice, and policy stakeholders. Prioritizing interdisciplinary collaboration among researchers across translational stages and fields (i.e., addiction, pain) could help address translational lags in early-stage pain research. Developing collaborative initiatives between clinicians and researchers across pain and addiction could help address issues related to fragmented care for individuals with chronic pain and SUD. Despite efforts to remove barriers to treatment access, disparities persist in the uptake of evidence-based treatment for addiction (e.g., buprenorphine). Addressing, and not just identifying, gaps in representation among underserved populations must remain a central focus of HEAL’s priorities. Finally, focusing on the effective implementation of evidence-based interventions in nonclinical settings, and engaging with community leaders and policy stakeholders to ensure these innovations reach all people equitably are essential steps forward. To mitigate the evolving overdose crisis, HEAL must continue to innovate and foster partnerships across research, policy, and public health sectors. Findings from this portfolio analysis could help the NIH connect researchers more efficiently with each other, better understand program uptake, and create material that increases the adoption and use of their findings.
